# Risk Factors for Whole Carcass Condemnations in the Swiss Slaughter Cattle Population

**DOI:** 10.1371/journal.pone.0122717

**Published:** 2015-04-22

**Authors:** Flavie Vial, Sara Schärrer, Martin Reist

**Affiliations:** 1 Veterinary Public Health Institute, Vetsuisse Faculty, University of Bern, Liebefeld, Switzerland; 2 Federal Food Safety and Veterinary Office, Liebefeld, Switzerland; University of Louisville, UNITED STATES

## Abstract

We used meat-inspection data collected over a period of three years in Switzerland to evaluate slaughterhouse-level, farm-level and animal-level factors that may be associated with whole carcass condemnation (WCC) in cattle after slaughter. The objective of this study was to identify WCC risk factors so they can be communicated to, and managed by, the slaughter industry and veterinary services. During meat inspection, there were three main important predictors of the risk of WCC; the slaughtered animal's sex, age, and the size of the slaughterhouse it was processed in. WCC for injuries and significant weight loss (visible welfare indicators) were almost exclusive to smaller slaughterhouses. Cattle exhibiting clinical syndromes that were not externally visible (e.g. pneumonia lesions) and that are associated with fattening of cattle, end up in larger slaughterhouses. For this reason, it is important for animal health surveillance to collect data from both types of slaughterhouses. Other important risk factors for WCC were on-farm mortality rate and the number of cattle on the farm of origin. This study highlights the fact that the many risk factors for WCC are as complex as the production system itself, with risk factors interacting with one another in ways which are sometimes difficult to interpret biologically. Risk-based surveillance aimed at farms with reoccurring health problems (e.g. a history of above average condemnation rates) may be more appropriate than the selection, of higher-risk animals arriving at slaughter. In Switzerland, the introduction of a benchmarking system that would provide feedback to the farmer with information on condemnation reasons, and his/her performance compared to the national/regional average could be a first step towards improving herd-management and financial returns for producers.

## Introduction

Veterinarians”need to emphasise the four pillars of the veterinary role—animal health, animal welfare, public health and the environment” [1]. Meat inspection at slaughterhouses, performed by trained veterinarians, contributes to the first three of these pillars. When whole or partial carcasses, are judged unfit for human consumption, they are condemned (judged to be unfit for human consumption) and removed from the food production chain. Condemnations at slaughterhouses are not just important for food safety; they are also of great interest to the farm animal production industry and also to veterinary services. First, their relevance is economic. Condemnations, and in particular whole carcass condemnations (WCC), result in substantial economic losses for producers. A full carcass condemnation equates to an on farm death with asset discounts resulting from transport costs and inflated disposal costs at the abattoir. Pig condemnations at slaughter in the Republic of Ireland in 2010 were estimated to result in an economic loss of more than five per cent of the net margin for pig production [2]. The identification of condemnation risk factors, either at the animal-level or at the farm-level, could have the potential to alleviate this problem, if the risk factors identified could be managed by producers. Meat inspection is an expensive process for public health authorities. We estimated the total costs of meat inspection in Switzerland between 2009 and 2011 to be 17.3 million Swiss francs (≈ 17.9 million US dollars). As there is considerable interest in moving towards risk-based surveillance at slaughterhouses, modernizing meat inspection, and making meat inspection more cost efficient, knowledge of these risks could also be useful to the slaughtering industry and national veterinary services. Finally, to be cost-effective, surveillance systems for rare and emerging infectious diseases should more fully exploit infrastructures in which potential health information carriers are currently available e.g. slaughterhouse or milk quality testing laboratories [3]. Currently in Switzerland, producers receive a report from the slaughterhouse after a WCC of one of their animals. However, no active discussion is undertaken on whether producers can decrease future condemnation risk by changing some of their herd management activities. The identification of factors associated with WCC in Switzerland is a prerequisite for the implementation of an animal health surveillance system based on meat inspection data; and will contribute to improving herd-management and financial returns to producers.

The literature on condemnation risks at slaughter has mostly focused on poultry and swine [4,5]. Recently, some studies have reported condemnation risk factors in sub-populations of slaughtered cattle. These studies tend to be geographically restricted to one region within a country [6,7]), focus on a small sample size of mainly dairy farms [7], or on a subset of animals taken to specific slaughterhouses [6,8]. In Switzerland, a slaughterhouse catchment area can extend well beyond its geographic location as a result of its reputation and its management practices. We have shown that slaughterhouses located in different regions differ in their WCC rate [9], so limiting a study on condemnation risks to one region may not be representative of the whole slaughter population.

The Swiss cattle population is heterogeneous in management practices and breeds. Small scale farming is predominant (mean herd size = 40) and two thirds of farms focus on dairy production [10]. Meat production occurs in several different management systems. Veal production is based on calves that are a by-product of the dairy industry. Calves are fattened either on their farm of origin, or on specialised fattening operations. In fattening operations, calves from many different farms of origin are gathered and mixed together. This practice challenges the immune systems of calves, increases their exposure to pathogens and thus facilitates disease occurrence[11]. Beef production (age 6 months to 2 years) consists of intensive fattening operations but also of suckler calves and young bulls from extensive mother-cow herds. The slaughter population under the age of 2 years has 3 times more male cattle than female cattle. The second largest quantity of beef production after veal calves is adult cattle. Adult slaughter cows are at the end of their productive cycle, i.e. dairy or mother cows and there is no upper limit in age (the mean age at slaughter during the study period for cows older than 2 years was 5.6 years and the oldest animal was 29). In this age category, only a few hundred bulls are slaughtered per month.

Some relevant animal-level condemnation risk factors, such as sex and age, have been identified by other studies [6,8] but we were particularly interested in animal movement data recoded in the Swiss National Cattle Register (NCR), compulsory under European Union Council Regulation (EC) No 1760/2000 of 17 July 2000. The different farm environments that cattle experience may result in different disease exposures during their lifetime (and hence potential WCC at slaughter). In this study, we used three years of meat-inspection data covering the whole of Switzerland to identify slaughterhouse-level, farm-level and animal-level factors that may be associated with an increased WCC risk in cattle. This paper summarises our findings based on >8000 WCC and discusses their potential implications for the routine use of slaughterhouse data for animal health surveillance and risk based meat inspection.

## Methods

### Study population

Our study population consisted of all slaughtered (n = 1’947’383) and condemned cattle in Switzerland between 01/01/2009 and 31/12/2011. A total of 9’951 WCC (3’049 and 6’902 from normal and emergency slaughters, respectively) were extracted from the Fleischkontrolldatenbank (FLEKO), the Swiss meat inspection database (for more details, see[9]). Emergency slaughter occurs when sick or injured animals are identified during ante-mortem inspection upon arrival at the slaughterhouse. These animals are kept separate from others and slaughtered last, in order to minimize cross-contamination with normally slaughtered carcasses. Swiss meat inspectors can chose among 44 WCC reasons as specified by legislation (817.190.1 Ordonnance du DFI du 23 novembre 2005 concernant l’hygiène lors de l’abattage d’animaux (OHyAb)). Some reasons relate to incidents that happen during transport to the slaughterhouse (e.g. dead on arrival) or improper slaughtering practices (e.g. soiled or heat-damaged carcasses). We excluded 535 WCC records with these reasons for WCC, as on-farm risk factors were unlikely to be relevant in such instances. Finally, we randomly selected 16’000 cattle that were slaughtered between 2009 and 2011 but not condemned.

### Risk factors

Risk factors for our study population were derived from the Swiss NCR which contains information on cattle holdings (e.g. location, production type), animals (e.g. birth date, sex, and breed), movement records (e.g. date, movement type) and stays (i.e. for every animal the start and end date of a stay on any holding is recorded). Each animal have an identification slaughtered number, served as a unique identifier to allow the tracing of condemned animals recorded in FLEKO database to the NCR database, which cover the entire Swiss cattle population The WCC risk factors were therefore rather broad, but they applied to a wide range of cattle holding types in Switzerland. Potential slaughter-level (1–3), animal-level (4–6) and farm-level (7–10) WCC risk factors were identified as follows:

1. Size of slaughterhouse (*S*_*s*_): Swiss slaughterhouses were ranked according to the volume of cattle processed between 2009 and 2011. Seven ranks were used: ranks 1 to 6 were the 1^st^ largest to 6^th^ largest, rank 7: included the 7^th^ largest and all the smaller slaughterhouses.
2. Month of slaughter (*S*_*m*_).
3. Year of slaughter (*S*_*y*_)
4. Sex of animal (*A*_*s*_): steers and non-castrated males were not differentiated.
5. Age at slaughter (*A*_*a*_): 1 = 0–180 days; 2 = 181–365 days; 3 = 366–730 days; 4 = > 730 days
6. Number of movements per year of the animal over its lifetime (*A*_*m*_).
7. Farm production type where the animal spent the greatest time on (*F*_*t*_).
8. Number of cattle per farm was defined as: All animals that shared some time in same herd with the animal of interest (*F*_*s*_).
9. Number of movements per farm was defined as: All arrivals during the time of stay for the animal of interest (*F*_*m*_).
10. Number of deaths per farm was defined as: All deaths during the time of stay of the animal of interest (*F*_*d*_).

A weighting was applied to the last three indicators (8–10) because animals will have spent different amounts of time at different farms. The last two indicators were also adjusted for herd size. So for example, if an animal lived a total of 365 days, in which time it spent 120 days on farm A (herd size = 50), 200 days on farm B (herd size = 35) and 45 days on farm C (herd size = 95), we used the following equation:

$$F_{d}=\left(\left(\frac{120*F_{dA}}{50}\right)+\left(\frac{200*F_{dB}}{35}\right)+\left(\frac{45*F_{dC}}{95}\right)\right)$$

Spearman’s rank correlation coefficient was calculated as a measure of statistical dependence between variables. Adjusting the last two indicators by herd size helped to deal with collinearity:

- Correlation between *F*_*s*_and *F*_*m*_: spearman rho = 0.78 (before) and rho = 0.36 (after correction)
- Correlation between *F*_*s*_ and *F*_*d*_: spearman rho = 0.66 (before) and rho = 0.36 (after correction)
- Correlation between *F*_*d*_ and *F*_*m*_: spearman rho = 0.51 (before) and rho = 0.06 (after correction)

### Statistical analyses

We first considered all WCC following normal and emergency slaughters separately. Each variable was evaluated for statistical significance using logistic regression with the outcome condemned/not condemned. For continuous variables, the model assumption of linearity between the predictor and the logit of y (outcome) was checked by plotting the observed proportions against mean predicted probabilities (points should be approximately on a straight line). Another method to test the assumption of linearity in the logit is to use the Box-Tidwell transformation. This involves adding a term of the form (X)ln(X) to the equation. If the coefficient for this variable is statistically significant, there is evidence of nonlinearity in the relationship between logit(Y) and X. In cases for which evidence of non-linearity was highlighted, the variable was modelled using restricted cube splines instead [12].

Analyses were performed in R version 3.1.1[13] using the packages {car} [14], {ggplot2}[15], {languageR}[16], {rms}[17], {faraway} [18] {effect}[19] and {MBESS}[20]. Variables with a p-value lower than 0.20 were included in a multivariable model in a second stage [21]. Interactions were also evaluated. A backward step-by-step procedure was used to select the final model excluding the non-significant variables (p > 0.05). For comparison of nested models, likelihood ratio tests and the Akaïke information criterion (AIC) were used. Bootstraping was used to validate the best models, and the area under the curve (AUC) was examined as a measure of predictive performance. Goodness of the fit of the final model was assessed using the le Cessie-van Houwelingen-Copas-Hosmer unweighted sum of squares test; and plots of residuals were examined.

Finally, this process was repeated (still separately for normal and emergency slaughters) using subsets of the WCC data, i.e. investigating potential WCC risk factors for specific reasons (e.g. sarcosporidiosis). Only condemnation reasons which totalled a minimum of 100 WCC over the three years period were further investigated.

## Results

Theoretically, the unique animal identifier should allow the traceability of 100% of cattle slaughtered in Switzerland. However, we found that around 6% of WCC could not be accurately traced back to the NCR (likely as a result of typos in the unique identifier number either by meat inspector data entry error or error in the NCR). Out of the original 9’416 WCC records from FLEKO, we could trace 8’818 animals to the NCR (2837 normal slaughters and 5982 emergency slaughters). Complete animal history (no missing values for any of the variables investigated) was available for 2’753 normal slaughtered cattle, 5’827 emergency slaughtered cattle and 15’895 slaughtered but not condemned cattle.

### WCC in normal slaughters

**Fig 1:**
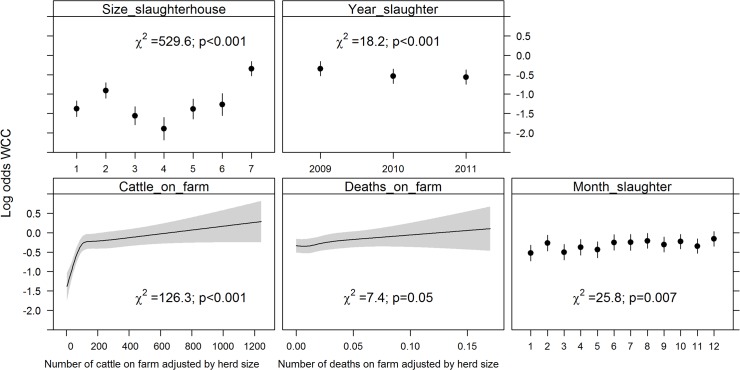
log (odds) for variables retained in the final model for WCC in normal slaughters. Only the main effects (and not the interaction terms) are represented.Footnote: Month of slaughter from 1 (January) to 12 (December); slaughterhouse size from 1to 6 (1^st^ largest to 6^th^ largest) and 7(from 7^th^ largest to the smallest).

**Fig 2:**
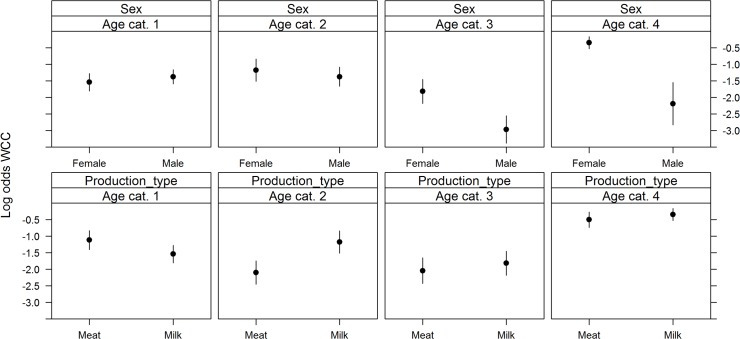
Significant interactions between age at slaughter and sex (top) and age at slaughter and production type (bottom) on the log (odds) of WCC in normal slaughters. Footnote: Age at slaughter cat. 1 (0–180 days); cat. 2 (181–365 days); cat. 3 (366–730 days); and cat. 4 (>730 days).

**Fig 3:**
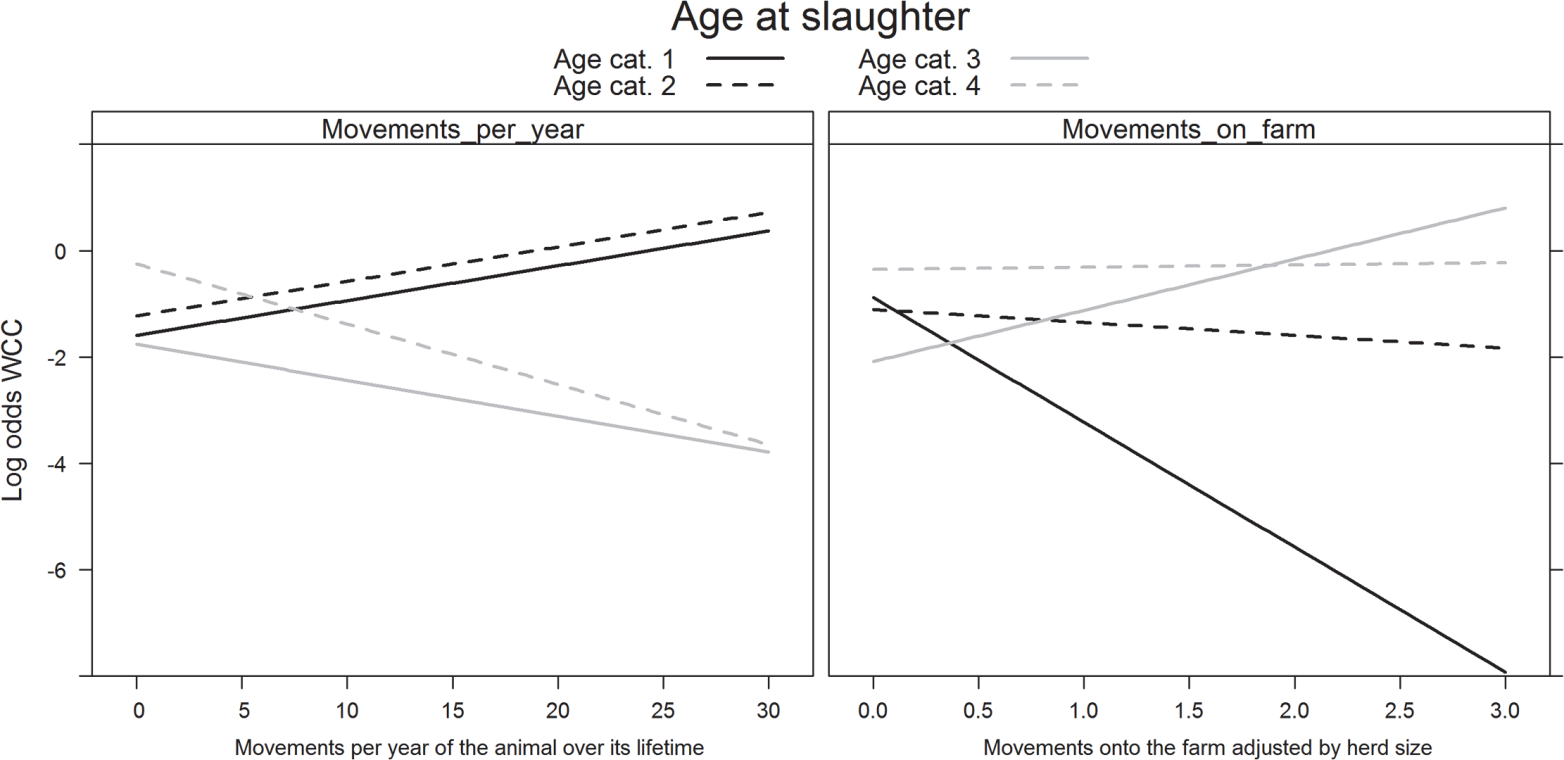
Significant interactions between age at slaughter and movements per year (left) and age at slaughter and movements on farm (right) on the log (odds) of whole carcass condemnations in normal slaughters. Footnote: Age at slaughter cat. 1 (0–180 days); cat. 2 (181–365 days); cat. 3 (366–730 days); and cat. 4 (>730 days).

**Fig 4:**
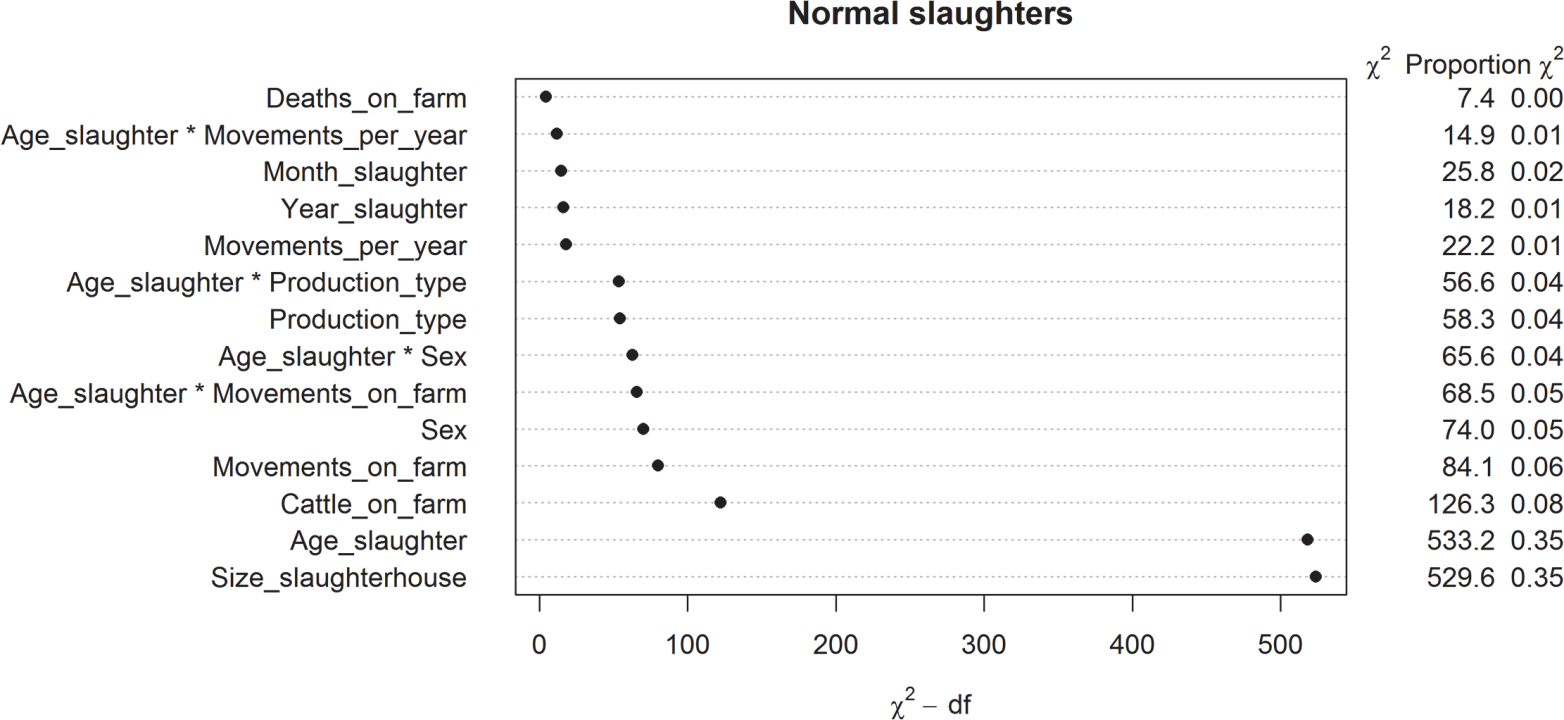
Risk factors for whole carcass condemnations following normal slaughter ranked by importance (top: lowest importance; bottom: highest importance)

All variables were significant at the 0.20 level when screened with univariable models (Table 1). Variables *A*_*m*_ and *F*_*s*_ showed evidence of non-linearity with the logit(Y) and were modelled using restricted cubic splines. All variables added to the multivariable logistic regression model were retained after backward selection (Fig 1). The risk of WCC varied with month of slaughter (χ2 = 25.84, df = 11, p = 0.01), as the risk was highest in December and lowest in January; and with year of slaughter (χ2 = 18.17, df = 2, p<0.001)(risk was lower in 2009 than in 2010 or 2011). The risk of WCC also varied with slaughterhouse size (χ2 = 529.63, df = 6, p<0.001), with a higher risk of WCC being found in smaller slaughterhouses. The number of deaths per farm was a significant WCC risk factor (χ2 = 7.45, df = 1, p = 0.05); as was the number of cattle per farm (χ2 = 126.33, df = 4, p<0.001). Age at slaughter significantly interacted with sex (χ^2^ = 65.62, df = 3, p<0.001) and production type (χ^2^ = 56.56, df = 3, p<0.001)(Fig 2). Dairy cows had a higher probability of being condemned than beef cows, except in the first age category (1–180 days). Females had a higher probability of being condemned than males, except in the first age category (1–180 days). Surprisingly, we found that the number of movements per farm (χ^2^ = 84.07, df = 4, p<0.001) appeared to decrease the risk of WCC for animals in the first age category (1–180 days); and that the animal’s movements per year (χ^2^ = 14.85, df = 3, p = 0.002) decreased the risk of WCC for the animals in the latter two age categories (>366 days) (Fig 3). The variables age at slaughter and slaughterhouse size explained a large proportion (70%) of the variability in the probability of a carcass being condemned (Fig 4).

**Table 1:** Distributions of carcass condemnation in normal slaughtered cattle (n = 2’753) compared to a selection of slaughtered cattle (n = 15’895) in Switzerland, recorded in the FLEKO meat inspection database between 2009–2011.

Variables	Classes	Outcome of carcass inspection		Odds ratio (OR)	95% confidence interval (CI)	P-value (Chi-square)	Akaike information criterion (AIC)
		Condemned N (%)	Not condemned N (%)				
None							15613
Sex of animal (*A*_*s*_)	Female	1’868 (68%)	7’611 (48%)	Reference		<0.001	15233
	Male	885 (32%)	8’284 (52%)	0.44	[0.40–0.47]		
Age at slaughter (*A*_*a*_)	0–180	834 (30%)	5’480 (34%)	Reference		<0.001	14648
	181–365	208 (8%)	2’498 (16%)	0.55	[0.47–0.64]		
	366–730	123 (4%)	3’048 (19%)	0.27	[0.22–0.32]		
	>730	1’588 (58%)	4’869 (31%)	2.14	[1.96–2.35]		
Size of slaughterhouse (*S*_*s*_)^φ^	1	391 (14%)	3’556 (22%)	Reference		<0.001	14994
	2	491 (18%)	2’519 (16%)	1.77	[1.54–2.04]		
	3	191 (7%)	2’401 (15%)	0.72	[0.60–0.87]		
	4	80 (3%)	1’211 (8%)	0.60	[0.47–0.77]		
	5	127 (5%)	857 (5%)	1.35	[1.09–1.66]		
	6	93 (3%)	797 (5%)	1.06	[0.83–1.34]		
	7	1’380 (50%)	4’554 (29%)	2.76	[2.45–3.11]		
Production type (*F*_*t*_)	Meat	617 (22%)	5’888 (37%)	Reference		<0.001	15380
	Milk	2’136 (78%)	10’007 (63%)	2.04	[1.85–2.24]		
Month of slaughter (*S*_*m*_)	Jan.	201 (7%)	1’413 (9%)	Reference		<0.001	15600
	Feb.	214 (8%)	1’218 (8%)	1.24	[1.00–1.52]		
	Mar.	214 (8%)	1’497 (9%)	1.00	[0.82–1.23]		
	Apr.	210 (8%)	1’310 (8%)	1.13	[0.92–1.39]		
	May	195 (7%)	1’336 (8%)	1.03	[0.83–1.27]		
	Jun.	225 (8%)	1’259 (8%)	1.26	[1.02–1.54]		
	Jul.	205 (7%)	1’150 (7%)	1.25	[1.02–1.55]		
	Aug.	250 (9%)	1’246 (8%)	1.41	[1.15–1.73]		
	Sep.	232 (8%)	1’231 (8%)	1.33	[1.08–1.63]		
	Oct.	269 (10%)	1’392 (9%)	1.36	[1.12–1.66]		
	Nov.	262 (10%)	1’466 (9%)	1.26	[1.03–1.53]		
	Dec.	276 (10%)	1’377 (9%)	1.41	[1.16–1.72]		
Year of slaughter (*S*_*y*_)	2009	981 (36%)	5’297 (33%)	Reference		0.06	15611
	2010	887 (32%)	5’284 (33%)	0.91	[0.82–1.00]		
	2011	885 (32%)	5’314 (33%)	0.90	[0.81–0.99]		
Number of movements per year of life (*A*_*m*_)		Min = 0	Min = 0	0.93	[0.90–0.96]	<0.001	15588
		Max = 28.08	Max = 38.42				
		Median = 0.48	Median = 0.86				
Number of cattle on farm (*F*_*s*_)		Min = 0	Min = 0	1.33	[1.19–1.49]	<0.001	15605*
		Max = 2’721.99	Max = 1’659.81				
		Median = 101.17	Median = 89				
Number of movements per farm (*F*_*m*_)		Min = 0	Min = 0	0.65	[0.53–0.80]	<0.001	15613
		Max = 2.95	Max = 4.01				
		Median = 0.27	Median = 0.28				
Number of deaths per farm (*F*_*d*_)		Min = 0	Min = 0	1.11	[1.10–1.21]	<0.001	15602*
		Max = 0.30	Max = 0.25				
		Median = 0.01	Median = 0.01				

* the relationship between the outcome and the independent variable was found to be non-linear and subsequently modelled using restricted cubic splines

^φ^ (1 to 6: 1^st^ largest to 6^th^ largest, 7: from 7^th^ largest to the smallest)

### WCC in emergency slaughters

**Fig 5:**
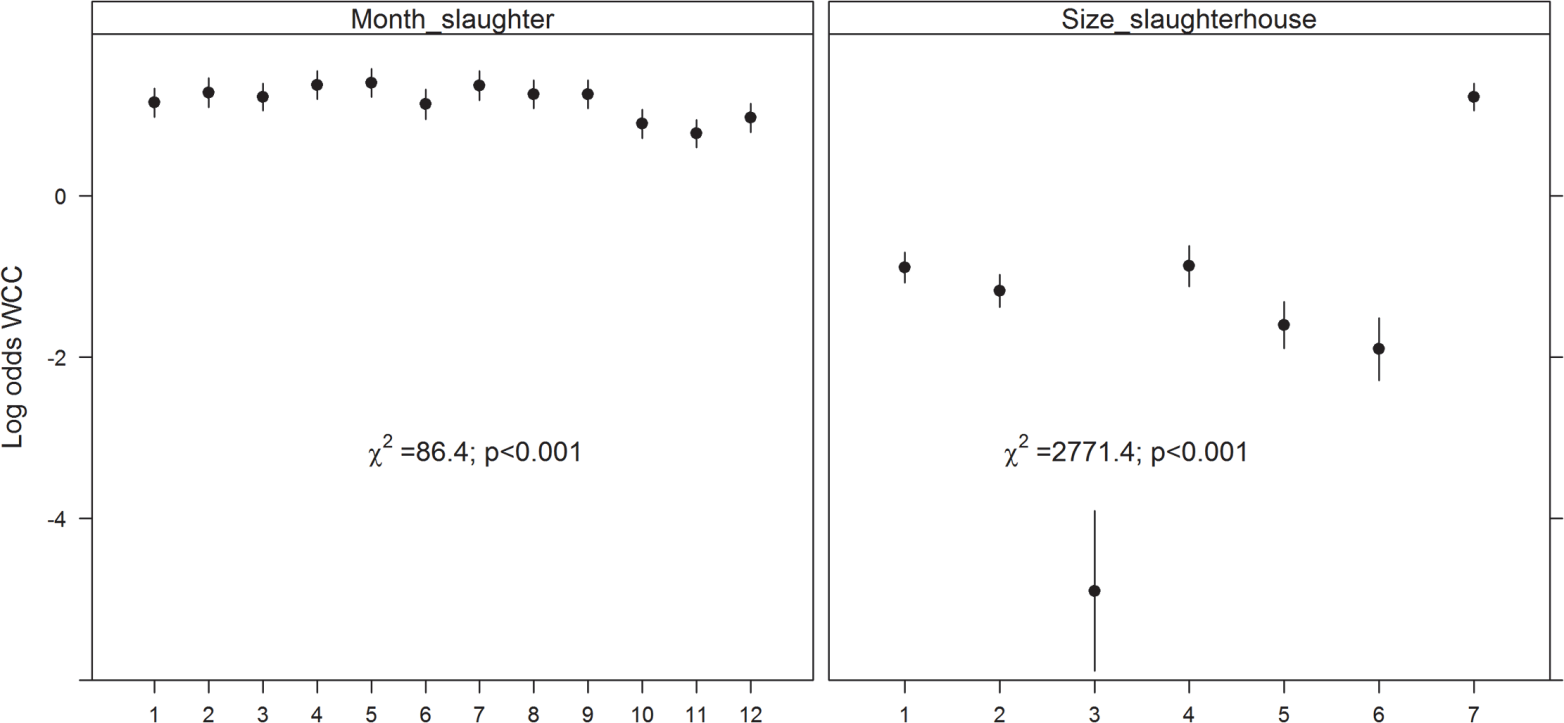
Log (odds) for variables retained in the final model for whole carcass condemnations (WCC)in emergency slaughters. Only the main effects (and not the interaction terms) are represented.Footnote: Month of slaughter from 1 (January) to 12 (December);slaughterhouse size from 1to 6 (1^st^ largest to 6^th^ largest) and 7(from 7^th^ largest to the smallest).

**Fig 6:**
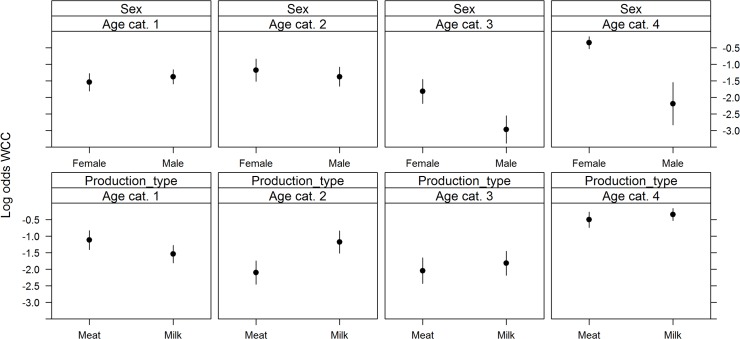
Significant interactions between age at slaughter and sex (top) and age at slaughter and production type (bottom) on the log (odds) of WCC in emergency slaughters. Footnote: Age at slaughter cat. 1 (0–180 days); cat. 2 (181–365 days); cat. 3 (366–730 days); and cat. 4 (>730 days).

**Fig 7:**
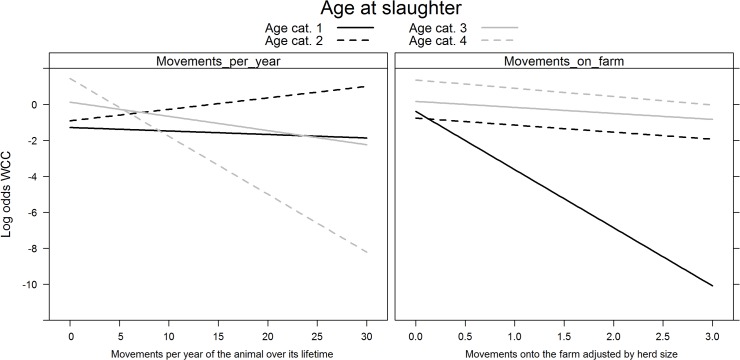
Significant interactions between age at slaughter and movements per year (left) and age at slaughter and movements on farm (right) on the log (odds) of WCC in emergency slaughters. Footnote: Age at slaughter cat. 1 (0–180 days); cat. 2 (181–365 days); cat. 3 (366–730 days); and cat. 4 (>730 days).

**Fig 8:**
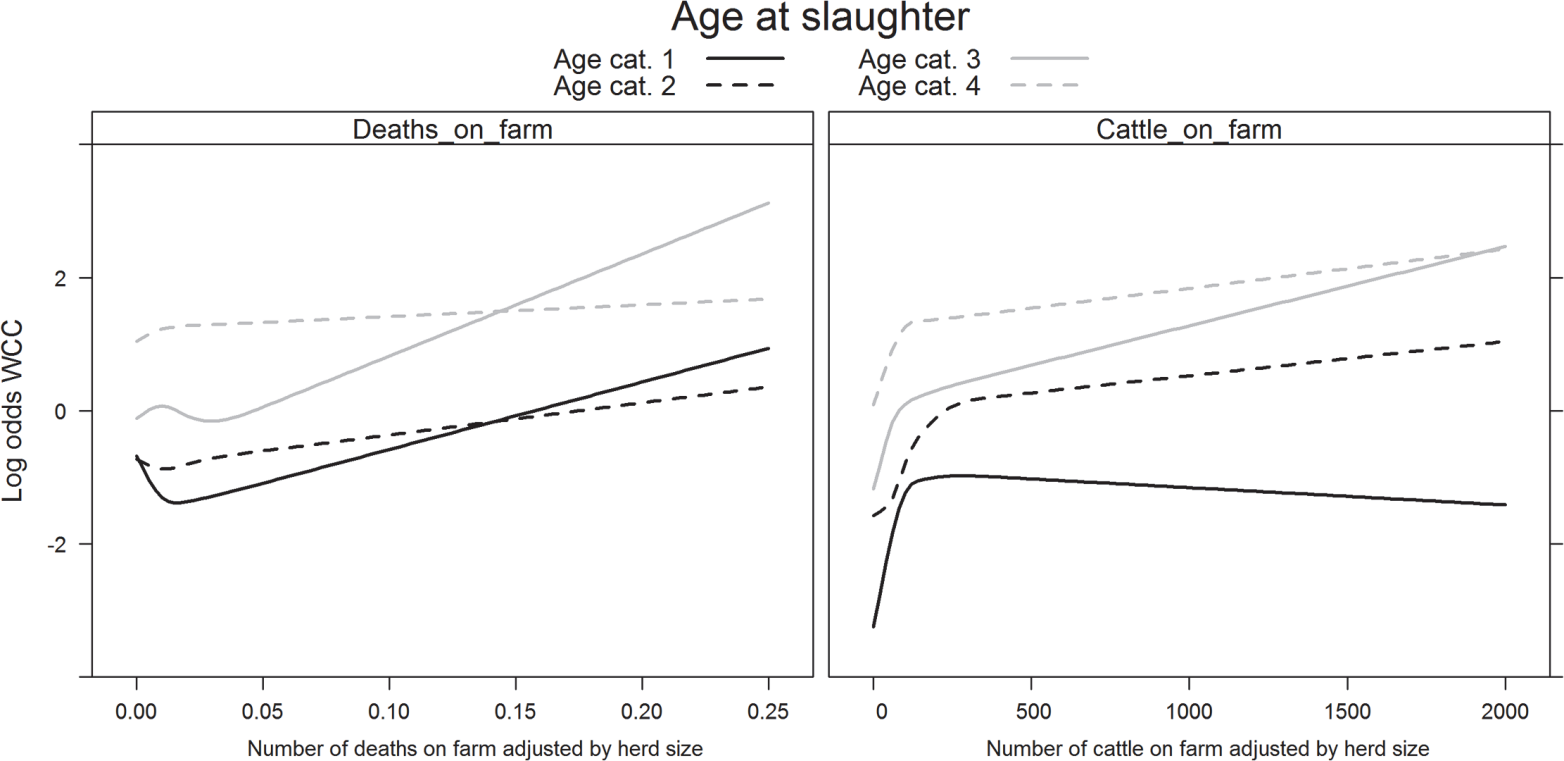
Significant interactions between age at slaughter and deaths on farm (left) and age at slaughter and number of cattle on farm (right) on the log (odds) of whole carcass condemnations (WCC) in emergency slaughters. Footnote: Age at slaughter cat. 1 (0–180 days); cat. 2 (181–365 days); cat. 3 (366–730 days); and cat. 4 (>730 days).

All variables were significant at the 0.20 level during the initial screening (Table 2), and the variables *A*_*m*_ and *F*_*s*_ once again showed evidence of non-linearity with the logit(Y). Year of slaughter was not retained in the multivariable logistic regression after backward selection. The risk of WCC varied with month of slaughter (χ^2^ = 88.44, df = 11, p<0.001) as the risk was highest in April/May and lowest between October and December. The risk of WCC also varied with slaughterhouse size (χ^2^ = 2771.37, df = 6, p<0.001), with a higher risk of WCC being found in smaller slaughterhouses (Fig 5). Age at slaughter significantly interacted with sex (χ^2^ = 121.65, df = 3, p<0.001) with males and females older than 366 days harbouring very different risks (Fig 6). As observed in normal slaughter, dairy cows had a higher probability of being condemned (χ^2^ = 58.64, df = 3, p<0.001), with the difference in risk being most prominent in individuals between the ages of 180–365 days. Age at slaughter also significantly interacted with the number of movements per farm (χ^2^ = 55.75, df = 3, p<0.001), with animals younger than 180 days showing the steepest decrease in WCC risk as the number of movements per farm increased. On the other hand, an increase in the animal’s number of movements per year (χ^2^ = 48.69, df = 3, p<0.001) was most strongly linked to a reduction in WCC in animals older than 730 days (Fig 7). The effects of the number of deaths on the farm (χ^2^ = 56.71, df = 9, p<0.001) and the number of cattle on farm (χ^2^ = 31.73, df = 12, p = 0.002) on WCC risk were also age-dependent (Fig 8). As with normal slaughters, the variables age at slaughter and slaughterhouse size explained over 80% of the variability in the probability of a carcass being condemned.

**Table 2:** Distributions of carcass condemnation in emergency slaughtered cattle (n = 5’827) compared to a selection of slaughtered cattle (n = 15’895) in Switzerland, recorded in the FLEKO meat inspection database between 2009–2011.

Variables	Classes	Outcome of carcass inspection		Odds ratio (OR)	95% confidence interval (CI)	P-value (Chi-square)	Akaike information criterion (AIC)
		CondemnedN (%)	Not condemned N (%)				
None							25265
Sex of animal (*A*_*s*_)	Female	5’013 (86%)	7’611 (48%)	Reference		<0.001	22449
	Male	814 (14%)	8’284 (52%)	0.15	[0.14–0.16]		
Age at slaughter (*A*_*a*_)	0–180	699 (12%)	5’480 (34%)	Reference		<0.001	21171
	181–365	264 (5%)	2’498 (16%)	0.83	[0.71–0.96]		
	366–730	292 (5%)	3’048 (19%)	0.75	[0.65–0.87]		
	>730	4’572 (78%)	4’869 (31%)	7.36	[6.74–8.05]		
Size of slaughterhouse (*S*_*s*_) ^φ^	1	514 (9%)	3’556 (22%)	Reference		<0.001	19657
	2	301 (5%)	2’519 (16%)	0.83	[0.71–0.96]		
	3	4 (<1%)	2’401 (15%)	0.01	[0.00–0.03]		
	4	128 (2%)	1’211 (8%)	0.73	[0.59–0.89]		
	5	76 (1%)	857 (5%)	0.61	[0.47–0.78]		
	6	35 (<1%)	797 (5%)	0.30	[0.21–0.43]		
	7	4’769 (82%)	4’554 (29%)	7.24	[6.55–8.02]		
Production type (*F*_*t*_)	Meat	1’216 (21%)	5’888 (37%)	Reference		<0.001	24733
	Milk	4’611 (79%)	10’007 (63%)	2.23	[2.08–2.40]		
Month of slaughter (*S*_*m*_)	Jan.	489 (8%)	1’413 (9%)	Reference		<0.001	25221
	Feb.	451 (8%)#Table2_ft2	1’218 (8%)	1.07	[0.92–1.24]		
	Mar.	573 (10%)	1’497 (9%)	1.11	[0.96–1.27]		
	Apr.	537 (9%)	1’310 (8%)	1.18	[1.03–1.37]		
	May	546 (9%)	1’336 (8%)	1.18	[1.02–1.36]		
	Jun.	421 (7%)	1’259 (8%)	0.97	[0.83–1.12]		
	Jul.	480 (8%)	1’150 (7%)	1.17	[1.04–1.40]		
	Aug.	505 (9%)	1’246 (8%)	1.21	[1.01–1.36]		
	Sep.	528 (9%)	1’392 (9%)	1.24	[1.07–1.43]		
	Oct.	420 (7%)	1’231 (8%)	0.87	[0.75–1.01]		
	Nov.	435 (7%)	1’466 (9%)	0.86	[0.74–0.99]		
	Dec.	442 (8%)	1’377 (9%)	0.93	[0.80–1.08]		
Year of slaughter (*S*_*y*_)	2009	1’830 (31%)	5’297 (33%)	Reference		0.008	25260
	2010	1’934 (33%)	5’284 (33%)	1.06	[0.98–1.14]		
	2011	2063 (35%)	5’314 (33%)	1.12	[1.04–1.21]		
Number of movements per year of life (*A*_*m*_)		Min = 0	Min = 0	0.63	[0.62–0.65]	<0.001	24131
		Max = 18.14	Max = 38.4				
		Median = 0.27	Median = 0.86				
Number of cattle on farm (*F*_*s*_)		Min = 0	Min = 0	1.64	[1.51–1.78]	<0.001	25229*
		Max = 2’410.07	Max = 1’659.81				
		Median = 107	Median = 89				
Number of movements per farm (*F*_*m*_)		Min = 0	Min = 0	0.63	[0.53–0.73]	<0.001	25231
		Max = 3.12	Max = 4.01				
		Median = 0.27	Median = 0.28				
Number of deaths per farm (*F*_*d*_)		Min = 0	Min = 0	1.15	[1.12–1.18]	0.001	25183*
		Max = 0.37	Max = 0.25				
		Median = 0.01	Median = 0.01				

* the relationship between the outcome and the independent variable was found to be non-linear and subsequently modelled using restricted cubic splines

^φ^ (1 to 6: 1^st^ largest to 6^th^ largest, 7: from 7^th^ largest to the smallest)

### Reasons for WCC: specific risk factors

**Fig 9:**
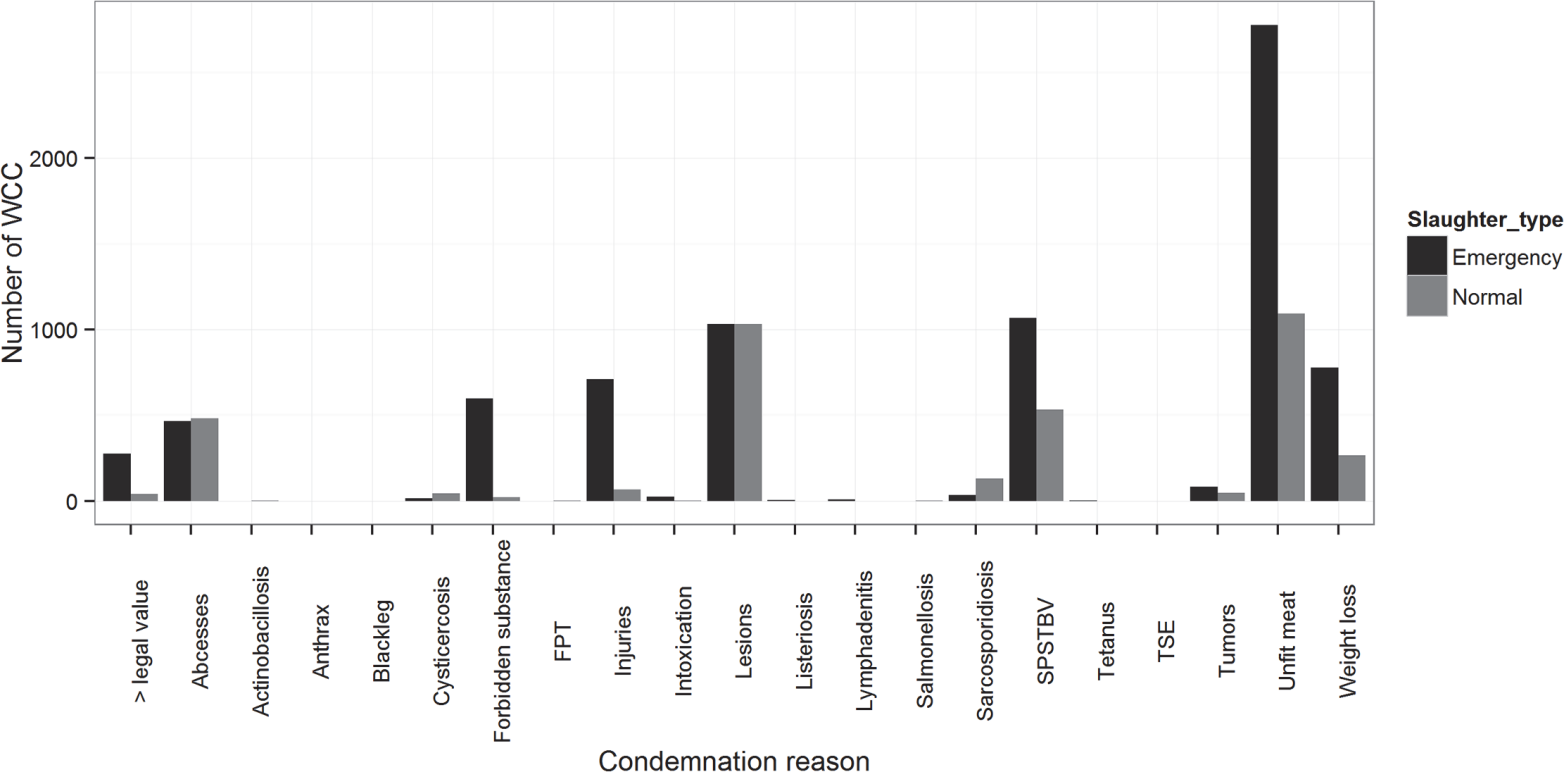
Whole carcass condemnation reasons in normal and emergency slaughters. Footnote: FPT: forbidden physical treatment; SPSTBV; symptoms of pyaemia, septicemia, toxemia, bacteremia or viremia; TSE: Transmissible spongiform encephalopathies.

While 44 possible reasons for condemnations exist in the legislation, most cattle WCC fall under 7 broad types of condemnations (Fig 9): 1)severe injuries; 2)symptoms of pyaemia, septicemia, toxemia, bacteremia or viremia; 3)acute lesions; 4)pronounced weight loss; 5)abscesses; 6)meat unfit for consumption (colour, consistency etc.); and 7)sarcosporidiosis. The risk factors identified in the analyses presented above remained valid when looking at specific WCC reasons (Table 3). The animal’s sex, age at slaughter and the slaughterhouse size were significant in all 12 models run. Interestingly, WCC for pronounced weight loss and severe injuries (potential animal welfare indicators) were uncommon in the 6 largest slaughterhouses and mostly recorded at smaller slaughterhouses. The next most commonly encountered risk factors (sequentially) were: The number of deaths per farm (11/12); farm production type (10/12); the interactions between sex and age at slaughter (10/12) and production type and age at slaughter (10/12). Differences in WCC between years (9/12) were more pronounced than between months (7/12), with an increase in WCC for many condemnations reasons over the three years of the study. The number of movements per farm and the animal’s number of movements per year of life were identified as risk factors in 75% of the models tested. The effect of the former often depended on the animal’s age at slaughter (significant interaction in 6/12 models). Other tested variables and interaction terms were seldom retained.

**Table 3:** Variables retained in the final multivariable models of the probability of WCC for a particular reason in normal (N) and emergency (E) slaughters.

Condemnation type	Slaughter type	*A*_*s*_	*F*_*t*_	*A*_*a*_	*S*_*s*_	*S*_*y*_	*S*_*m*_	*F*_*s*_	*A*_*m*_	*F*_*m*_	*F*_*d*_	*A*_*a*_ * *A*_*s*_ ^c^	*A*_*a*_ *F_*t*_	*A*_*a*_ * *A*_*m*_	*A*_*a*_ * *F*_*m*_	*A*_*a*_ * *F*_*d*_
Severe injuries	E (n = 709)	x		x	x ^a^	x			x		x	x		x		
Symptoms of pyaemia, septicemia,toxemia, bacteremia or viremia ^a^	E (n = 1’069)	x	x	x	x	x	x		x^b^	x^b^	x	x	x			
	N (n = 535)	x	x	x	x	x	x			x	x		x		x	
Acute lesions	E (n = 1’034)	x	x	x	x		x		x^b^	x	x	x	x		x	
	N (n = 1’031)	x	x	x	x	x	x	x		x	x	x	x		x	
Pronounced weight loss	E (n = 779)	x	x	x	x^a^			x^b^	x^b^	x^b^	x	x	x			x
	N (n = 267)	x	x	x	x ^a^	x	x		x^b^	x	x	x	x		x	x
Abcesses	E (n = 465)	x	x	x	x	x		x^b^	x^b^	x^b^	x	x	x			
	N (n = 482)	x	x	x	x						x	x	x			
Meat unfit for consumption	E (n = 2’323)	x	x	x	x	x	x		x^b^	x	x	x	x		x	x
	N (n = 934)	x	x	x	x	x	x		x^b^	x	x	x	x		x	x
Sarcosporidiosis	N (n = 132)	x		x	x^a^	x										
Total (/12)		12	10	12	12	9	7	3	8	9	11	10	10	1	6	4

Only condemnation reasons that resulted in at least 100 whole carcass condemnations between 2009–2011 in each type of slaughter were investigated. Size of slaughterhouse (S_s_); month of slaughter (S_m_); year of slaughter (S_y_); sex of animal (A_s_); age at slaughter (A_a_); number of movements per year of the animal over its lifetime (A_m_); production type of the farm (F_t_); number of cattle on farm (F_s_); number of movements per farm (F_m_); and number of deaths per farm (F_d_).

^a^ Because too few condemnations were recorded in the largest slaughterhouses, the slaughterhouses were ranked as follows: The 6 largest slaughterhouses versus all others

^b^ the relationship between the log(Y) and the independent variable was found to be non-linear and subsequently modelled using restricted cubic splines

^c^ “*” denotes an interaction between variables

## Discussion

The three main important predictors of the risk of WCC in Swiss cattle were the slaughtered animal's sex, age, and the size of the slaughterhouse it was processed in. The same three variables were identified as risk factors for offal, partial and whole carcass condemnations in a recent study based on ten French cattle slaughterhouses [8]. Other important risk factors for WCC that we identified were on-farm mortality rate and the number of cattle on the farm of origin. Case-control studies to identify risk factors for specific types of condemnations at slaughter may bring additional insight to our findings based on NCR data. For example, Flutsch *et al*. [22] reported that railways or a car park close to grazing areas for cattle is a risk factor for the occurrence of bovine cysticercosis recorded in cattle at meat inspection in Switzerland. However, even if only evaluating broad risk factor categories for WCC, our study highlights the fact that such risk factors already interact with one another in complex ways.

The effect of the interaction between age at slaughter and the animal’s sex on WCC risk can be explained by the Swiss production system. Male calves in Switzerland have been shown to suffer higher levels of mortality and unwanted early slaughter than their female counterparts [11]. The nursing of male calves might be neglected because they are sold within a few weeks after birth because of their low economic value. Therefore, slaughtered male calves (<180 days) may be more likely to result in a WCC. Older males are typically slaughtered because they are at the end of the production cycle (whether fattening or breeding bulls), while older females are typically slaughtered due to health issues (e.g. lameness, mastitis, fertility issues) or decreased milk production. The risk of a WCC therefore, is expected to become higher in females older than 180 days. Age and sex have also been identified as factors associated with bovine cysticercosis recorded in cattle at meat inspection in Denmark [23]

Similarly, we expected an interaction between animal age at slaughter and production type. Breeding for increased production has had negative side effects on health and fertility traits in dairy cattle [24]. They are at a higher risk of production diseases (e.g. mastitis, reduced fertility) with increasing age, than beef cattle. For example, the incidence of metabolic and nutritional diseases progressively increases over years of milk production in dairy cows [25]. In a Spanish study, organic calves had fewer organs condemnations at slaughter when compared with intensive and to a lesser extent with conventional calves [26].

Our original hypothesis was that the greater the number of farm environments an animal experienced, the higher the disease exposure during its lifetime (and hence potential WCC at slaughter). This seems to be the case for younger cattle (<1 year), however it is less clear why animals older than 1 year (normal slaughters) were at a lower risk of WCC when they were moved between more farms. A possible explanation could be that individuals of lower health were not moved to alpine pastures in the summer, but were kept on the farm all year long, therefore reducing their potential exposure to pathogens during mixing of cattle from multiple farms on alpine pastures. No satisfactory biological explanation could be found to explain how the number of movements per farm could decrease the risk of WCC in animals slaughtered before the age of 6 months. This finding is counter-intuitive and upon closer examination of the data, it seems that this effect is mainly driven by data coming from the dairy calves (results not presented).

Animals coming from larger herds were generally more at risk of being condemned at slaughter than animals from smaller herds. We found, however, that calves (<6months) coming from larger herds had a slightly reduced risk of being condemned following emergency slaughter. One possible explanation could be that emergency slaughters of calves were more likely to be the result of animals arriving at the slaughterhouse injured during transport rather than animals arriving sick. Animals suffering from a fresh injury may be at a lower risk of condemnation than sick animals. The general health status of calves in large farms is usually good but the risk of injury during transport may be heightened when large numbers of calves are loaded onto a truck. Animals coming from farms with higher mortality rates were also at an increased risk of WCC. Animal death on farms may be linked to diseases or to poor farm management. Either or both of which may increase the probability of condemnation at slaughter of herd members that are shipped together.

Since the size of the slaughterhouse an animal is brought to was identified as one of the top three risk factors for post-mortem condemnation, should we focus surveillance on larger slaughterhouses where the risk of WCC is higher? A word of caution is necessary as higher WCC rates may not necessarily be due to animals of lower health. Large slaughterhouses tend to be affiliated with major retailers who only accept animals of very high certain quality. Interestingly, a French study established that the odds of a condemnation at slaughter (whether partial or whole) were at least twice as great for farmers who did not adhere to the quality charter of an international retailer [7]. It was also interesting to note that WCC for injuries and weight loss (visible welfare indicators) were almost exclusive to smaller slaughterhouses (as these animals are not accepted for slaughter by the larger slaughterhouses). It may be that animals with lesions that are not visible on the living animal (e.g. pneumonia) but associated with fattening (environment with high infectious pressure, high density of animals and mixing of animals from many farms) still end up in the larger slaughterhouses while animals exhibiting external signs of poor health don’t. For these reasons it would be important for the surveillance to monitor data from both types of slaughterhouses.

The purpose of meat inspection is to ensure food safety and to detect epizooties [27]. The key to the abandonment of incision and palpation (traditional meat inspection practices) is the ability to categorise livestock and carcases according to risk, based on verifiable food chain information which also includes information about the production system and animal age category components [28,29]. A feasibility study to evaluate the practicality of sampling in the slaughterhouse and to assess the possibility of selecting animals or farms according to given risk factors has taken place in Switzerland. The identification of specific descriptors that could represent risk factors is very difficult at the slaughter plant, as at present only very limited information can be extracted directly from documents accompanying individual animals. The only recognizable criterion from a carcass is calf versus adult, as the size of carcass and organs are visually distinguishable. Sex, breed or production types are no longer recognizable once the skin and sexual organs are removed. Because of there being no centralised data management system to enable real–time data exchange and because of missing technical aids for the identification of preselected animals, the possibilities for risk-based or even herd-level surveillance are very limited. However, with the future introduction of technological solutions that enable information exchanges between meat inspectors and the veterinary authorities [30], a risk-based selection of individuals at the slaughterhouse should soon be possible.

## Conclusion

The reasons to condemn a whole carcass as being unfit for human consumption are manifold. Risk based meat inspection should therefore be based on rather general criteria like ‘age at slaughter’, ‘emergency slaughter’ or the ‘size of the farm of origin’. However, this study shows that the risk factors for WCC are as complex as the production system itself. Many risk factors interact with one another in ways that difficult to explain biologically. A risk based animal health surveillance system, aimed at farms with reoccurring health problems, i.e. a history of above average condemnation rates, might therefore be more appropriate. Furthermore, changes in management practices could decrease the future risk of WCC. The introduction of a benchmarking system that would provide feedback to the farmer with information on the condemnation reasons, and their specific performance compared to the national/regional average would be a step towards improving herd-management and financial returns to producers.
